# Advancing How We Learn from Biodesign to Mitigate Risks with Next-Generation Genome Engineering

**DOI:** 10.34133/2020/9429650

**Published:** 2020-04-25

**Authors:** Paul E. Abraham, Jessy L. Labbé, Amber A. McBride

**Affiliations:** ^1^Chemical Sciences Division, Oak Ridge National Laboratory, Oak Ridge, Tennessee 37831, USA; ^2^Biosciences Division, Oak Ridge National Laboratory, Oak Ridge, Tennessee 37831, USA

## Abstract

In the last decade, the unprecedented simplicity and flexibility of the CRISPR-Cas system has made it the dominant transformative tool in gene and genome editing. However, this democratized technology is both a boon and a bane, for which we have yet to understand the full potential to investigate and rewrite genomes (also named “genome biodesign”). Rapid CRISPR advances in a range of applications in basic research, agriculture, and clinical applications pose new risks and raise several biosecurity concerns. In such a fast-moving field of research, we emphasize the importance of properly communicating the quality and accuracy of results and recommend new reporting requirements for results derived from next-generation genome engineering.

Following initial concerns about the possibility of unintended gene editing, [1–3] early assessments began reporting that CRISPR-Cas activity was reasonably specific, paving the way for several clinical studies [4, 5]. However, while CRISPR-Cas was poised to become the gene editing tool of choice in clinical contexts, new research began to show that initial assessments were too limited and missed a substantial proportion of genomic perturbations, which could have hazardous consequences in the clinical context [6]. As a response to the lack of certainty, most scientists emphasized the importance of the precautionary principle, advocating for more evaluation to ensure that any proposed application of CRISPR-Cas gene editing receives robust assessments to reduce uncertainty surrounding its outcomes and probabilities.

The inherent technology limitations of CRISPR-Cas gene editing methods have practical consequences that must be disseminated and discussed in useful ways. To provide the most valuable feedback and leverage prior works in subsequent designs, the exactness of the information and lessons learned should be intentionally communicated. Cartography is a convenient model to follow as a framework to communicate reliability and exactness [7, 8]. Maps are useful products of scientific knowledge because uncertainty is clearly defined by their conventionalized features and scales. For example, it is easy to recognize “borders of ignorance” because the information is explicitly represented as a blank spot. Through iterative revisions based on knowledge acquisition, maps are then improved with few, if any, blank spaces. Following this analogy, although the “border of ignorance” is often acknowledged for activity related to CRISPR-Cas systems, it is rarely clearly defined in publications. Consequentially, many users are likely unaware of potential hazards and their levels of functional risk.

How can CRISPR-Cas researchers improve their skills as “mapmakers” to help users avoid potential pitfalls or hazards and foster high confidence in both the possible outcomes and their respective probabilities? While experimental work will continue to improve our understanding of all the factors influencing CRISPR-Cas activity, it is only now becoming clear how well these factors can be generalized across different strategies or in diverse genetic backgrounds. As with any map, there is an inescapable limit of applicability that needs to be defined. Therefore, when charting a course for safer genome engineering, first and foremost, researchers must clearly define the purpose of their maps and the intended users. Also, researchers must be explicit about the quality of their results in terms of extensiveness and reliability. Recent reports point to a rich array of computation approaches for more effective biodesign and detection methods that highlight the different ways our current knowledge for decision-making and risk assessment are becoming more assertive, contestable, or uncertain [9–11]. Over the past decade, significant advancements have been made in our ability to answer the fundamental question of whether the engineered nuclease acted at genomic locations other than its intended site, illuminating our understanding of genome-wide editing activity [12–15]. We encourage researchers to implement and be more sensitive, rigorous, and comprehensive in detection approaches. Also, rigorous examination of DNA repair outcomes following genome editing activity demonstrates that outcomes are reproducible and predictable [4, 16]. On the other hand, reports highlighting incongruencies between detection methods emphasize the hazards and limitations of experimental designs developed for discovering CRISPR activity [6, 17]. Therefore, rather than being reduced to a single measurement, it is recommended that methodological “blind spots” be avoided by using an eyes-wide-open approach. We define methodological uncertainties to mean uncertainty that can arise when analytical methods give confounding evaluative results. By implementing more comprehensive and complementary screening methods, we encourage combination of anchored sequencing methods (e.g., AMPs-seq) with genome-wide activity profiling (e.g., CIRCLE-seq, Digenome-seq) that would increase the understanding of the frequency and location of unwanted outcomes, which will improve users’ roadmaps for future biodesign research endeavors.

Finally, more consideration to the management of newly released information would avoid distorted representations of CRISPR-Cas activity, which can negatively influence decision-making and policymaking processes. Recently, Schaefer et al. claimed that CRISPR-Cas nuclease activity resulted in genome-wide point mutations at an alarming frequency [18]. This report was ultimately retracted after several correspondences highlighted the many deficiencies in the study and found no evidence to support the proposed frequency of mutations. Interestingly, the rapid dissemination of comments and discussions on the accuracy and soundness of this publication primarily occurred outside traditional, peer-reviewed channels through preprint services and scientific blogs. Would a similar response have been made for less-developed research outside the more often explored mammalian systems?

Next-generation editing technologies are necessary in the comprehension of biological systems and industrial developments with fast innovation related to throughput and automation. This requires reliable studies with clear reports (including limitation and failure) to better facilitate future biodesign endeavors and foster safer research. Because reviews cannot catch all mistakes and limitations, we encourage specialists work with journals to establish and subsequently implement rigorous checklists of metrics to evaluate such reports. This would standardize the quality for learning purposes and implementation in other organisms or experiments. Addressing these issues will advance our ability to learn from next-generation genome engineering research and improve the safety and outcome of tomorrow’s biodesign.
